# Incomplete reporting of complex interventions: a call to action for journal editors to review their submission guidelines

**DOI:** 10.1186/s13063-023-07215-1

**Published:** 2023-03-22

**Authors:** Mairead Ryan, Tammy Hoffmann, Riikka Hofmann, Esther van Sluijs

**Affiliations:** 1 Medical Research Council Epidemiology Unit, University of Cambridge, Cambridge, UK; 2 Faculty of Education, University of Cambridge, Cambridge, UK; 3 Faculty of Health Sciences and Medicine, Bond University, Gold Coast, Australia

**Keywords:** Reporting checklists, Complex interventions, Journal editors, Reproducibility, Research integrity, Submission guidelines

## Abstract

Reporting of intervention research has been inadequate for many years. The development and promotion of freely available checklists aims to address this problem by providing researchers with a list of items that require reporting to enable study interpretation and replication. In this commentary, we present evidence from a recent systematic review of 51 randomised controlled trials published 2015–2020 that inadequate intervention reporting remains a widespread issue and that checklists are not being used to describe all intervention components. In 2022, we assessed the submission guidelines of 33 journals that published articles included in our review and found that just one at the time encouraged the use of reporting checklists for all intervention components. To drive progress, we contacted the editors of the other 32 journals and requested that they update their submission guidelines in response. We conclude by highlighting the waste associated with current practices and encourage journals from all fields to urgently review their submission guidelines. Only through collective action can we build an evidence base that is fit for purpose.

**Supplementary Information:**

The online version contains supplementary material available at 10.1186/s13063-023-07215-1.

## Reporting guidelines in research

Randomised controlled trials and other forms of intervention evaluations constitute a considerable proportion of public health research activity. For many years, researchers have typically provided inadequate descriptions of the intervention(s) they tested [1], which has limited the scientific and applied value of this activity [2]. From the 1990s, in response to evidence of poor reporting and its costs, bodies such as the International Committee of Medical Journal Editors (ICMJE) and the Enhancing the QUAlity and Transparency Of health Research (EQUATOR) Network began supporting the development and promotion of evidence-based reporting guidelines [3]. There now exists a library of freely available reporting guidelines to support researchers in providing sufficient detail to enable study interpretation and replication [4]. The publication of the Template for Intervention Description and Replication (TIDieR) checklist in 2014 [5] aimed to improve reporting standards in intervention research specifically. The checklist outlines a minimum set of items considered essential for intervention description and replication. We present evidence in this commentary that the authors of complex interventions mostly use such checklists to describe only one part of the overall intervention. We draw on our experience of reviewing school-based physical activity research to present evidence of underreporting on interventions targeted at change agents.

**Fig. 1:**
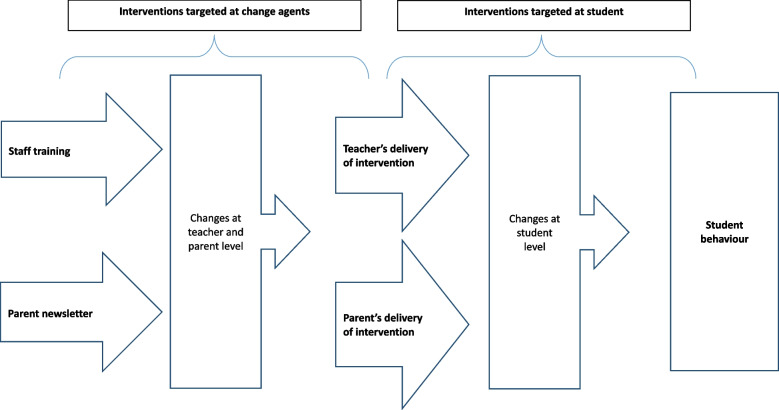
Schematic overview of multi-actor interventions

Successful implementation of school-based interventions is often dependent on behaviour change by key actors or ‘change agents’ in students’ lives (e.g. teachers, parents/guardians, peers). For example, school staff may be required to change their teaching practices, and/or parents may be required to implement changes. Figure 1, adapted from the Medical Research Council guidance on the development and evaluation of complex interventions [6], illustrates that change agent-targeted interventions (sometimes referred to as ‘implementation strategies’ [7]) (e.g. staff training programmes, parent newsletters) play an important role in the logic model of the overall intervention. Nevertheless, they are frequently overlooked as behaviour change interventions in and of themselves [7] and their impact on process evaluation outcomes (e.g. percentage of intended educational sessions delivered) and trial effectiveness outcomes (e.g. students’ behaviour) is poorly understood.

## Evidence of inadequate reporting on change agent interventions in school-based research

We recently completed a systematic review of school-based physical activity interventions to study if specific features of staff training programmes were associated with intervention fidelity and students’ study outcomes [8]. This was a significant research gap because the majority of children and adolescents worldwide are not sufficiently physically active [9, 10] and global intervention efforts, which largely focus on school settings, have mostly failed [11, 12]. Reasons for outcome failures were unknown as previous review efforts to address similar research questions were impeded by incomplete descriptions of interventions published 1999–2015 [13]. Given the greater availability and promotion of intervention reporting guidelines since 2015 (e.g. [5]), we solely reviewed interventions published since 2015, in anticipation that reporting quality had improved. We conducted a systematic, comprehensive and inclusive search for details on intervention descriptions of included randomised controlled trials, pooling information from multiple sources (e.g. protocols, process evaluations, outcome evaluations, trial registries, study websites; *n* = 183 in total). See Additional file 1 for details about methods and sources included. We aimed to identify descriptions of staff training programmes based on items in the TIDieR checklist [5]. Where incomplete reporting was identified, we contacted lead authors for further information.

We included 51 trials, reporting on 53 training programmes [8]. We found that, prior to contacting authors, complete information was only available for one of the training programmes we reviewed (2%). Basic details (e.g. use of theory, location of the intervention) were missing from the majority of interventions (see Additional file 2 for breakdown by TIDieR item before and after author contact). Descriptions across all available study sources largely focused on the intervention(s) aimed at the student, and while some study authors reported using checklists to describe student-targeted interventions, no checklists were reportedly used to describe change agent-targeted interventions.

At the time of writing this commentary, we checked the websites of all the journals that published articles included in our review (*n* = 33) and found that just one explicitly requested submission of reporting checklists for all intervention components. Our findings suggest that inadequate reporting remains a prevalent issue despite the availability of infrastructure (e.g. reporting checklists and online appendices/repositories to overcome manuscript word limits) and that current systems, including journal submission guidelines, are enabling wasteful practices to persist. Our findings are also particularly alarming given that adherence to reporting guidelines is reportedly better in larger and controlled studies [14]; the sample of publications we reviewed reported on medium-to-large-scale randomised controlled trials (median sample size: 779; interquartile range: 361–1397). While we found that the percentage of reported items improved considerably by contacting authors (see Additional file 2), this resource-intensive task is a poor replacement for Open Science practices. Moreover, readers of intervention outputs are not informed that they lack sufficient information to reliably replicate intervention(s) or interpret reported outcomes. As reliance upon change agents is common in many other intervention settings (e.g. hospitals, police custody suites, nursing homes [15–17]), our findings have wider relevance beyond school-based research.

## Action taken

Current reporting practices are stifling learning opportunities within and beyond public health and limiting the types of knowledge that can be gained from costly evaluations. Without complete descriptions of all interventions under evaluation, the scientific community has no means of interpreting study outcomes or replicating effective interventions [5, 18]. This is resulting in poor outcomes for members of the public who have both funded and/or participated in intervention studies. Research communities must now move beyond describing poor reporting practices to taking action and pay equal attention to reporting of all change agent-targeted interventions. Although we urge authors of intervention papers to provide complete descriptions of all intervention components, we recognise the important gatekeeper role that journal editors play in setting research communication and reporting standards [14]. We contacted the editors of the other 32 journals (see Additional file 3), inviting them to update their submission guidelines in response to our findings. Specifically, we asked that they require intervention authors to submit separate reporting checklists for each of the interventions that have been delivered within a study, including interventions targeted at change agents. To date, we have received a reply from 27 journals, 26% of whom have updated their submission guidelines in response, including ‘British Journal of Sports Medicine’, ‘Journal of Physical Activity and Health’, ‘Sport, Exercise, and Performance Psychology’, 'Journal of Science and Medicine in Sport' and ‘Journal of Experimental Social Psychology’ (see Additional file 3 for a complete list).

## Further action is needed

While we are pleased a number of journals have updated their guidelines, ongoing inaction from others will enable current practices to continue. The 2013 Declaration of Helsinki emphasises that journals have an ethical obligation to reject incomplete reports of research for publication [19]. We now call on all journals that publish intervention research, regardless of their field, to urgently review their submission policies. We also highlight the role that researchers, editorial teams, funders, and reviewers can all play in encouraging and supporting journals in this effort. Resources from the EQUATOR Network may be useful in guiding related discussion: https://www.equator-network.org/toolkits/using-guidelines-in-journals/creating-your-journals-reporting-guideline-policy/. As we approach the 10-year publication anniversary of the TIDieR intervention reporting guideline, we ask—how much more waste will be tolerated before action is taken?

## Supplementary Information

**Additional file 1. **Methods used to assess completeness of TIDieR checklist items in systematic review.

**Additional file 2. **List of TIDieR items reported before and after author contact.

**Additional file 3. **Complete list of journals contacted (*n*=32) and action taken (*n*=7).

## Data Availability

A summary of reviewed studies and their outputs is available in Additional file 1.

## Abbreviations

TIDieR: Template for Intervention Description and Replication
ICMJE: International Committee of Medical Journal Editors
EQUATOR: Enhancing the QUAlity and Transparency Of health Research
