# Supplementary material for: Incomplete reporting of complex interventions: a call to action for journal editors to review their submission guidelines
Source: Trials. 2023 Mar 22;24:176. doi: 10.1186/s13063-023-07215-1 (PMC10031932; doi:10.1186/s13063-023-07215-1)
Supplement: Supplementary file 1 — Additional file 1. Methods used to assess completeness of TIDieR checklist items in systematic review. [file 13063_2023_7215_MOESM1_ESM.docx]

**Additional file 1:** Methods used to assess completeness of TIDieR checklist items in systematic review [1]

After full-text screening, we conducted forward and backward citation tracking and searched through articles and their supplementary materials for any peer-reviewed publications (e.g. protocols, process evaluations, outcome evaluations) and other outputs (e.g. trial registries, study websites) relevant to the trials eligible for inclusion. As a trial may have more than one output, all study publications and outputs were pooled for data extraction. A full list of trial publications and outputs is listed below.

The pooled publications and outputs were then searched to complete the Template for Intervention Description and Replication (TIDieR) checklist [2], a reporting guideline of the minimum set of items considered essential for intervention description and replication (e.g. use of theory, duration, mode of delivery). All data extraction was conducted by a single reviewer. Where multiple training programmes were delivered within a study (e.g. in the form of content, dose, material etc. beyond local adaptation or personalisation), and outcome data were reported for each arm, data was sought and extracted for each arm.

If TIDieR items were not identified and perceived as applicable, then lead authors of included articles were contacted. They were requested to check and complete a partially filled TIDieR-based form, and to add any relevant study publications not listed. Authors were given three weeks to respond with a reminder email.

**References**

1. Ryan M, Alliott O, Ikeda E, Luan Ja, Hofmann R, van Sluijs E. Features of effective staff training programmes within school-based interventions targeting student activity behaviour: a systematic review and meta-analysis. *International Journal of Behavioral Nutrition and Physical Activity* 2022, 19(1):125.

2. Hoffmann TC, Glasziou PP, Boutron I, Milne R, Perera R, Moher D, Altman DG, Barbour V, Macdonald H, Johnston M *et al*. Better reporting of interventions: template for intervention description and replication (TIDieR) checklist and guide. *BMJ* 2014, 348:g1687.

**List of trial publications and outputs identified during data extraction period**

Leading author of article included in review, name of intervention (or relevant acronym)

Study outputs identified

**Aadland et al., ASK**

Aadland, K. N., Ommundsen, Y., Anderssen, S. A., Brønnick, K. S., Moe, V. F., Resaland, G. K., ... & Aadland, E. (2019). Effects of the Active Smarter Kids (ASK) physical activity school-based intervention on executive functions: a cluster-randomized controlled trial. *Scandinavian Journal of Educational Research*, *63*(2), 214-228.

Resaland, G. K., Aadland, E., Moe, V. F., Aadland, K. N., Skrede, T., Stavnsbo, M., ... & Kvalheim, O. M. (2016). Effects of physical activity on schoolchildren's academic performance: The Active Smarter Kids (ASK) cluster-randomized controlled trial. *Preventive Medicine*, *91*, 322-328.

Stavnsbo, M., Aadland, E., Anderssen, S. A., Chinapaw, M., Steene-Johannessen, J., Andersen, L. B., & Resaland, G. K. (2020). Effects of the Active Smarter Kids (ASK) physical activity intervention on cardiometabolic risk factors in children: A cluster-randomized controlled trial. *Preventive Medicine*, *130*, 105868.

Resaland, G. K., Moe, V. F., Aadland, E., Steene-Johannessen, J., Glosvik, Ø., Andersen, J. R., ... & Anderssen, S. A. (2015). Active Smarter Kids (ASK): Rationale and design of a cluster-randomized controlled trial investigating the effects of daily physical activity on children’s academic performance and risk factors for non-communicable diseases. *BMC public health*, *15*(1), 1-10.

<https://www.clinicaltrials.gov/ct2/show/NCT02132494?term=NCT02132494&draw=2&rank=1>

**Adab et al., WAVES**

**References:**

Adab, P., Pallan, M. J., Lancashire, E. R., Hemming, K., Frew, E., Barrett, T., ... & Daley, A. (2018). Effectiveness of a childhood obesity prevention programme delivered through schools, targeting 6 and 7 year olds: cluster randomised controlled trial (WAVES study). *bmj*, *360*.

Adab, P., Pallan, M. J., Lancashire, E. R., Hemming, K., Frew, E., Griffin, T., ... & Deeks, J. (2015). A cluster-randomised controlled trial to assess the effectiveness and cost-effectiveness of a childhood obesity prevention programme delivered through schools, targeting 6-7 year old children: the WAVES study protocol. *BMC Public Health*, *15*(1), 488.

Griffin, T. L., Clarke, J. L., Lancashire, E. R., Pallan, M. J., Passmore, S., & Adab, P. (2015). Teacher experiences of delivering an obesity prevention programme (The WAVES study intervention) in a primary school setting. *Health Education Journal*, *74*(6), 655-667.

Adab, P., Barrett, T., Bhopal, R., Cade, J. E., Canaway, A., Cheng, K. K., ... & Ekelund, U. (2018). The West Midlands ActiVe lifestyle and healthy Eating in School children (WAVES) study: a cluster randomised controlled trial testing the clinical effectiveness and cost-effectiveness of a multifaceted obesity prevention intervention programme targeted at children aged 6-7 years. *Health Technology Assessment (Winchester, England)*, *22*(8), 1.

Griffin, T. L., Clarke, J. L., Lancashire, E. R., Pallan, M. J., Adab, P., & WAVES study trial investigators. (2017). Process evaluation results of a cluster randomised controlled childhood obesity prevention trial: the WAVES study. *BMC Public Health*, *17*(1), 681.

<http://www.isrctn.com/ISRCTN97000586>

**Aittasalo et al., KIDS OUT!**

**References:**

Aittasalo, M., Jussila, A. M., Tokola, K., Sievänen, H., Vähä-Ypyä, H., & Vasankari, T. (2019). Kids Out; evaluation of a brief multimodal cluster randomized intervention integrated in health education lessons to increase physical activity and reduce sedentary behavior among eighth graders. *BMC public health*, *19*(1), 415.

Jussila, A. M., Vasankari, T., Paronen, O., Sievänen, H., Tokola, K., Vähä-Ypyä, H., ... & Aittasalo, M. (2015). KIDS OUT! Protocol of a brief school-based intervention to promote physical activity and to reduce screen time in a sub-cohort of Finnish eighth graders. *BMC public health*, *15*(1), 634.

<https://clinicaltrials.gov/ct2/show/NCT01633918?term=NCT01633918&rank=1>

**Anderson et al., AFLY5**

**References:**

Anderson, E. L., Howe, L. D., Kipping, R. R., Campbell, R., Jago, R., Noble, S. M., ... & Lawlor, D. A. (2016). Long-term effects of the Active for Life Year 5 (AFLY5) school-based cluster-randomised controlled trial. *BMJ open*, *6*(11).

Lawlor, D. A., Jago, R., Noble, S. M., Chittleborough, C. R., Campbell, R., Mytton, J., ... & Kipping, R. R. (2011). The Active for Life Year 5 (AFLY5) school based cluster randomised controlled trial: study protocol for a randomized controlled trial. *Trials*, *12*(1), 181.

Kipping, R. R., Howe, L. D., Jago, R., Campbell, R., Wells, S., Chittleborough, C. R., ... & Lawlor, D. A. (2014). Effect of intervention aimed at increasing physical activity, reducing sedentary behaviour, and increasing fruit and vegetable consumption in children: active for Life Year 5 (AFLY5) school based cluster randomised controlled trial. *Bmj*, *348*, g3256.

Lawlor, D. A., Kipping, R. R., Anderson, E. L., Howe, L. D., Chittleborough, C. R., Moure-Fernandez, A., ... & Jago, R. (2016). Active for Life Year 5: a cluster randomised controlled trial of a primary school-based intervention to increase levels of physical activity, decrease sedentary behaviour and improve diet.

Campbell, R., Rawlins, E., Wells, S., Kipping, R. R., Chittleborough, C. R., Peters, T. J., ... & Jago, R. (2015). Intervention fidelity in a school-based diet and physical activity intervention in the UNITED KINGDOM: Active for Life Year 5. *International Journal of Behavioral Nutrition and Physical Activity*, *12*(1), 141.

<http://www.isrctn.com/ISRCTN50133740>

**Belton et al., Y-PATH**

**References:**

Belton, S., McCarren, A., McGrane, B., Powell, D., & Issartel, J. (2019). The Youth-Physical Activity Towards Health (Y-PATH) intervention: Results of a 24 month cluster randomised controlled trial. *PloS one*, *14*(9), e0221684.

Belton, S., O’Brien, W., Meegan, S., Woods, C., & Issartel, J. (2014). Youth-physical activity towards health: Evidence and background to the development of the Y-PATH physical activity intervention for adolescents. *BMC Public Health*, *14*(1), 122.

McGrane, B., Belton, S., Fairclough, S. J., Powell, D., & Issartel, J. (2018). Outcomes of the Y-PATH Randomized controlled trial: can a school-based intervention improve fundamental movement skill proficiency in adolescent youth?. *Journal of Physical Activity and Health*, *15*(2), 89-98.

Belton, S., O’Brien, W., McGann, J., & Issartel, J. (2019). Bright spots physical activity investments that work: Youth-Physical Activity Towards Health (Y-PATH). *British journal of sports medicine*, *53*(4), 208-212.

<http://www.isrctn.com/ISRCTN20495704>

**Bundy et al., Sydney Playground Project**

**References:**

Bundy, A., Engelen, L., Wyver, S., Tranter, P., Ragen, J., Bauman, A., ... & Perry, G. (2017). Sydney playground project: a cluster‐randomized trial to increase physical activity, play, and social skills. *Journal of school health*, *87*(10), 751-759.

Bundy, A. C., Naughton, G., Tranter, P., Wyver, S., Baur, L., Schiller, W., ... & Niehues, A. (2011). The Sydney playground project: popping the bubblewrap-unleashing the power of play: a cluster randomized controlled trial of a primary school playground-based intervention aiming to increase children's physical activity and social skills. *BMC public health*, *11*(1), 680.

Niehues, A. N., Bundy, A., Broom, A., Tranter, P., Ragen, J., & Engelen, L. (2013). Everyday uncertainties: reframing perceptions of risk in outdoor free play. *Journal of Adventure Education & Outdoor Learning*, *13*(3), 223-237.

Bundy, A. C., Naughton, G., Tranter, P., Wyver, S., Baur, L., Schiller, W., . . . Brentnall, J. (2011). The Sydney playground project: popping the bubblewrap - unleashing the power of play: a cluster randomized controlled trial of a primary school playground-based intervention aiming to increase children's physical activity and social skills. *BMC Open Access, 11*, 1-9.

Bundy, A. C., Wyver, S., Beetham, K. S., Ragen, J., Naughton, G., Tranter, P., . . . Sterman, J. (2015). The Sydney playground project - levelling the playing field: A cluster trial of a primary school-based intervention aiming to promote manageable risk-taking in children with disability. *BMC Public Health*, 1-6.

Engelen, L., Bundy, A. C., Naughton, G., Simpson, J. M., Bauman, A., Ragen, J., . . . van der Ploeg, H. P. (2013). Increasing physical activity in young primary school children - it's child's play: A cluster randomised controlled trial. *Preventive Medicine, 56*(5), 319-325.

Grady-Dominguez, P., Ihrig, K., Lane, S., Aberle, J., Beetham, K., Ragen, J., . . . Bundy, A. (2020). Reframing risk: Working with caregivers of children with disabilities to promote risk-taking in play. International Review of Research in Developmental Disabilities, 59, 1-45. doi:https://doi.org/10.1016/bs.irrdd.2020.09.001

Niehues, A. N., Bundy, A., Broom, A., & Tranter, P. (2015). Parents' perception of risk and the influence on children's everday activities. *Journal of Child and Family Studies, 24*, 809-820.

Niehues, A., Bundy, A., Broom, A., & Tranter, P. (2016). Reframing healthy risk taking: Parents’ dilemmas and strategies to promote children’s well-being. *Journal of Occupational Science, 23*(4), 449-463.

Sterman, J., Villenueve, M., Spencer, G., Wyver, S., Beetham, K., Naughton, G., . . . Bundy, A. (2020). Creating play opportunities on the school playground: Educator experiences of the Sydney Playground Project. *Occupational Therapy Journal of Australia, 67*(1), 62-73.

<http://www.anzctr.org.au/Trial/Registration/TrialReview.aspx?ACTRN=12611000089932>

**Chan et al., A+FMS**

**References:**

Chan, C. H., Ha, A. S., Ng, J. Y., & Lubans, D. R. (2019). The A+ FMS cluster randomized controlled trial: An assessment-based intervention on fundamental movement skills and psychosocial outcomes in primary schoolchildren. *Journal of Science and Medicine in Sport*, *22*(8), 935-940.

Chan, C., Ha, A., & Ng, J. Y. (2016). Improving fundamental movement skills in Hong Kong students through an assessment for learning intervention that emphasizes fun, mastery, and support: the A+ FMS randomized controlled trial study protocol. *SpringerPlus*, *5*(1), 724.

<https://www2.ccrb.cuhk.edu.hk/registry/public/318>

**Christiansen et al., SPACE**

**References:**

Christiansen, L. B., Toftager, M., Pawlowski, C. S., Andersen, H. B., Ersbøll, A. K., & Troelsen, J. (2017). Schoolyard upgrade in a randomized controlled study design—how are school interventions associated with adolescents’ perception of opportunities and recess physical activity. *Health Education Research*, *32*(1), 58-68.

Toftager, M., Christiansen, L. B., Kristensen, P. L., & Troelsen, J. (2011). SPACE for physical activity-a multicomponent intervention study: study design and baseline findings from a cluster randomized controlled trial. *BMC Public Health*, *11*(1), 777.

Toftager, M., Christiansen, L. B., Ersbøll, A. K., Kristensen, P. L., Due, P., & Troelsen, J. (2014). Intervention effects on adolescent physical activity in the multicomponent SPACE study: a cluster randomized controlled trial. *PLoS One*, *9*(6), e99369.

*Space - rum til fysisk aktivitet. Samlet evaluering af en helhedsorienteret, forebyggende indsats for børn og unge, januar 2014. Af Jens Troelsen (red.), Lars Breum Christiansen, Mette Toftager, Else Olesen, Betina Højgaard, Anne Brøcker, Stinne Aaløkke Ballegaard, Lone Grøn, Louise Ladegaard, Thomas Tjørnelund Nielsen og Brian Linke* <https://www.sdu.dk/sif/-/media/images/sif/udgivelser/2014/space_rum_til_fysisk_aktivitet.pdf>

<http://www.isrctn.com/ISRCTN79122411?q=ISRCTN79122411&filters=&sort=&offset=1&totalResults=1&page=1&pageSize=10&searchType=basic-search>

<http://www.cirhp.dk/> - not accessible

[http://www.forebyggelsescenter.dk/side.asp?side=8&id=12&ver=United Kingdom](http://www.forebyggelsescenter.dk/side.asp?side=8&id=12&ver=uk) - not accessible

**Cohen et al., SCORES**

**References:**

Cohen, K. E., Morgan, P. J., Plotnikoff, R. C., Callister, R., & Lubans, D. R. (2015). Physical activity and skills intervention: SCORES cluster randomized controlled trial. *Medicine and science in sports and exercise*, *47*(4), 765-774.

Lubans, D. R., Morgan, P. J., Weaver, K., Callister, R., Dewar, D. L., Costigan, S. A., ... & Plotnikoff, R. C. (2012). Rationale and study protocol for the supporting children’s outcomes using rewards, exercise and skills (SCORES) group randomized controlled trial: A physical activity and fundamental movement skills intervention for primary schools in low-income communities. *BMC public health*, *12*(1), 427.

Cohen, K. E., Morgan, P. J., Plotnikoff, R. C., Hulteen, R. M., & Lubans, D. R. (2017). Psychological, social and physical environmental mediators of the SCORES intervention on physical activity among children living in low-income communities. *Psychology of Sport and Exercise*, *32*, 1-11.

<https://www.anzctr.org.au/Trial/Registration/TrialReview.aspx?id=343424>

**Donnelly et al., A + PACC**

**References:**

Donnelly, J. E., Hillman, C. H., Greene, J. L., Hansen, D. M., Gibson, C. A., Sullivan, D. K., ... & Herrmann, S. D. (2017). Physical activity and academic achievement across the curriculum: results from a 3-year cluster-randomized trial. *Preventive medicine*, *99*, 140-145.

Donnelly, J. E., Greene, J. L., Gibson, C. A., Sullivan, D. K., Hansen, D. M., Hillman, C. H., ... & Herrmann, S. D. (2013). Physical activity and academic achievement across the curriculum (A+ PAAC): rationale and design of a 3-year, cluster-randomized trial. *BMC public health*, *13*(1), 1-8.

Szabo-Reed, A. N., Willis, E. A., Lee, J., Hillman, C. H., Washburn, R. A., & Donnelly, J. E. (2019). The influence of classroom physical activity participation and time on task on academic achievement. *Translational Journal of the American College of Sports Medicine*, *4*(12), 84-95.

Szabo-Reed, A. N., Willis, E. A., Lee, J., Hillman, C. H., Washburn, R. A., & Donnelly, J. E. (2017). Impact of 3 years of classroom physical activity bouts on time-on-task behavior. *Medicine and science in sports and exercise*, *49*(11), 2343.

<https://clinicaltrials.gov/ct2/show/NCT01699295>

**Drummy et al., Unnamed**

**References:**

Drummy, C., Murtagh, E. M., McKee, D. P., Breslin, G., Davison, G. W., & Murphy, M. H. (2016). The effect of a classroom activity break on physical activity levels and adiposity in primary school children. *Journal of paediatrics and child health*, *52*(7), 745-749.

**Duncan et al., Healthy Homework study**

**References:**

Duncan, S., Stewart, T., McPhee, J., Borotkanics, R., Prendergast, K., Zinn, C., ... & Schofield, G. (2019). Efficacy of a compulsory homework programme for increasing physical activity and improving nutrition in children: a cluster randomised controlled trial. *International Journal of Behavioral Nutrition and Physical Activity*, *16*(1), 80.

[*http://www.anzctr.org.au/Trial/Registration/TrialReview.aspx?ACTRN=12618000590268*](http://www.anzctr.org.au/Trial/Registration/TrialReview.aspx?ACTRN=12618000590268)

Duncan, S., McPhee, J. C., Schluter, P. J., Zinn, C., Smith, R., & Schofield, G. (2011). Efficacy of a compulsory homework programme for increasing physical activity and healthy eating in children: the healthy homework pilot study. *International Journal of Behavioral Nutrition and Physical Activity*, *8*(1), 127.

**Dyrstad et al., the Active School Study**

**References:**

Dyrstad, S. M., Kvalø, S. E., Alstveit, M., & Skage, I. (2018). Physically active academic lessons: acceptance, barriers and facilitators for implementation. *BMC public health*, *18*(1), 1-11.

Kvalø, S. E., Bru, E., Brønnick, K., & Dyrstad, S. M. (2017). Does increased physical activity in school affect children's executive function and aerobic fitness?. *Scandinavian journal of medicine & science in sports*, *27*(12), 1833-1841.

Seljebotn, P. H., Skage, I., Riskedal, A., Olsen, M., Kvalø, S. E., & Dyrstad, S. M. (2019). Physically active academic lessons and effect on physical activity and aerobic fitness. The Active School study: A cluster randomized controlled trial. *Preventive Medicine Reports*, *13*, 183-188.

<https://clinicaltrials.gov/ct2/show/NCT03436355?term=NCT03436355&rank=1>

<https://www.activesmarterkids.com/>

Skage, I., Ertesvåg, S.K., Roland, P. & Dyrstad, S.M. (2020). Implementation of physically active lessons: A 2-year follow-up. Evaluation and Program Planning, 83, 101874.

Skage, I. & Dyrstad, S.M. (2019). “It`s not because we don`t believe in it …”: Headteachers` perceptions of implementing physically active lessons in school. *BMC Public Health*, 19, 1674.

**Escriva-Boulley et al., Unnamed**

**References:**

Escriva-Boulley, G., Tessier, D., Ntoumanis, N., & Sarrazin, P. (2018). Need-supportive professional development in elementary school physical education: Effects of a cluster-randomized control trial on teachers’ motivating style and student physical activity. *Sport, Exercise, and Performance Psychology*, *7*(2), 218.

**Filho et al., Fortaleça sua Saúde**

**References:**

Barbosa Filho, V. C., da Silva, K. S., Mota, J., Beck, C., & da Silva Lopes, A. (2016). A physical activity intervention for brazilian students from low human development index areas: a cluster-randomized controlled trial. *Journal of Physical Activity and Health*, *13*(11), 1174-1182.

Barbosa Filho, V. C., da Silva Lopes, A., Lima, A. B., de Souza, E. A., do Amaral Gubert, F., Silva, K. S., ... & Mota, J. (2015). Rationale and methods of a cluster-randomized controlled trial to promote active and healthy lifestyles among Brazilian students: the “Fortaleça sua Saúde” program. *BMC Public Health*, *15*(1), 1212.

Araújo, Thábyta & Carvalho, Queliane & Barbosa Filho, Valter & Costa, Ana & Gubert, Fabiane & Vieira, Neiva. (2016). Educação em saúde no ambiente escolar - estudo de intervenção com professores da rede pública. Revista Tendências da Enfermagem Profissional - ReTEP. 8. 2024.

Souza, Evanice & Castro, Ângela & Sousa, Antônia & Alves, Felipe. (2016). Physical Education classes in the Empower your Health Project: an analysis from the standpoint of Teachers. Journal of Physical Activity and Health. 21. 198-206.

Lopes, Iraneide & Linard, Jair & Silva, Magna & Barbosa Filho, Valter. (2020). IMPLEMENTAÇÃO DO PROGRAMA DE PROMOÇÃO DO ESTILO DE VIDA ATIVO EM ESTUDANTES: O "FORTALEÇA SUA SAÚDE. 31. 14. 10.4025/jphyseduc.v31i1.3125.

<https://clinicaltrials.gov/ct2/show/NCT02439827>

**Gray et al., Choice Control and Change**

**References:**

Gray, H. L., Contento, I. R., & Koch, P. A. (2015). Linking implementation process to intervention outcomes in a middle school obesity prevention curriculum,‘Choice, Control and Change’. *Health education research*, *30*(2), 248-261.

Contento, I. R., Koch, P. A., Lee, H., & Calabrese-Barton, A. (2010). Adolescents demonstrate improvement in obesity risk behaviors after completion of choice, control & change, a curriculum addressing personal agency and autonomous motivation. *Journal of the American Dietetic Association*, *110*(12), 1830-1839.

Lee, H., Contento, I. R., & Koch, P. (2013). Using a systematic conceptual model for a process evaluation of a middle school obesity risk-reduction nutrition curriculum intervention: choice, control & change. *Journal of Nutrition Education and Behavior*, *45*(2), 126-136.

**Ha et al., SELF-FIT**

**References:**

Ha, A. S., Lonsdale, C., Lubans, D. R., & Ng, J. Y. (2020). Increasing Students' Activity in Physical Education: Results of the Self-determined Exercise and Learning For FITness Trial. *Medicine and Science in Sports and Exercise*, *52*(3), 696-704.

Ha, A. S., Lonsdale, C., Lubans, D. R., & Ng, J. Y. (2018). Increasing students’ physical activity during school physical education: rationale and protocol for the SELF-FIT cluster randomized controlled trial. *BMC Public Health*, *18*(1), 1-12.

<https://www.anzctr.org.au/Trial/Registration/TrialReview.aspx?id=368560>

**Ha et al., Unnamed**

Ha, A. S., Lonsdale, C., Ng, J. Y., & Lubans, D. R. (2017). A school-based rope skipping program for adolescents: Results of a randomized trial. *Preventive medicine*, *101*, 188-194.

Ha, A. S., Lonsdale, C., Ng, J. Y., & Lubans, D. R. (2014). A school-based rope skipping intervention for adolescents in Hong Kong: protocol of a matched-pair cluster randomized controlled trial. *BMC Public Health*, *14*(1), 1-8.

<https://www.anzctr.org.au/Trial/Registration/TrialReview.aspx?id=364862>

**Harrington et al., Girls Active**

**References:**

Harrington, D. M., Davies, M. J., Bodicoat, D. H., Charles, J. M., Chudasama, Y. V., Gorely, T., ... & Edwards, R. T. (2018). Effectiveness of the ‘Girls Active’school-based physical activity programme: A cluster randomised controlled trial. *International journal of behavioral nutrition and physical activity*, *15*(1), 40.

Edwardson, C. L., Harrington, D. M., Yates, T., Bodicoat, D. H., Khunti, K., Gorely, T., ... & Davies, M. J. (2015). A cluster randomised controlled trial to investigate the effectiveness and cost effectiveness of the ‘Girls Active’intervention: a study protocol. *BMC Public Health*, *15*(1), 526.

Gorely, T., Harrington, D. M., Bodicoat, D. H., Davies, M. J., Khunti, K., Sherar, L. B., ... & Edwardson, C. L. (2019). Process evaluation of the school-based Girls Active programme. *BMC public health*, *19*(1), 1187.

<http://www.isrctn.com/ISRCTN10688342>

**Have et al., Unnamed**

**References:**

Have, M., Nielsen, J. H., Ernst, M. T., Gejl, A. K., Fredens, K., Grøntved, A., & Kristensen, P. L. (2018). Classroom-based physical activity improves children’s math achievement-A randomized controlled trial. *PloS one*, *13*(12), e0208787.

Have, M., Nielsen, J. H., Gejl, A. K., Ernst, M. T., Fredens, K., Støckel, J. T., ... & Kristensen, P. L. (2016). Rationale and design of a randomized controlled trial examining the effect of classroom-based physical activity on math achievement. *BMC Public Health*, *16*(1), 304.

<https://clinicaltrials.gov/ct2/show/NCT02488460>

**Hillman et al., A + PACC**

**References:**

Donnelly, J. E., Hillman, C. H., Greene, J. L., Hansen, D. M., Gibson, C. A., Sullivan, D. K., ... & Herrmann, S. D. (2017). Physical activity and academic achievement across the curriculum: results from a 3-year cluster-randomized trial. *Preventive medicine*, *99*, 140-145.

Donnelly, J. E., Greene, J. L., Gibson, C. A., Sullivan, D. K., Hansen, D. M., Hillman, C. H., ... & Herrmann, S. D. (2013). Physical activity and academic achievement across the curriculum (A+ PAAC): rationale and design of a 3-year, cluster-randomized trial. *BMC public health*, *13*(1), 1-8.

Szabo-Reed, A. N., Willis, E. A., Lee, J., Hillman, C. H., Washburn, R. A., & Donnelly, J. E. (2019). The influence of classroom physical activity participation and time on task on academic achievement. *Translational Journal of the American College of Sports Medicine*, *4*(12), 84-95.

Szabo-Reed, A. N., Willis, E. A., Lee, J., Hillman, C. H., Washburn, R. A., & Donnelly, J. E. (2017). Impact of 3 years of classroom physical activity bouts on time-on-task behavior. *Medicine and science in sports and exercise*, *49*(11), 2343.

<https://clinicaltrials.gov/ct2/show/NCT01699295>

**Hodges et al., KIA**

**References**

Hodges, M. G., Kulinna, P. H., Van Der Mars, H., & Lee, C. (2016). Knowledge in action: Fitness lesson segments that teach health-related fitness in elementary physical education. *Journal of teaching in physical education*, *35*(1), 16-26.

**Hollis et al., PA4E1**

**References:**

Hollis, J. L., Sutherland, R., Campbell, L., Morgan, P. J., Lubans, D. R., Nathan, N., ... & Cohen, K. E. (2016). Effects of a ‘school-based’physical activity intervention on adiposity in adolescents from economically disadvantaged communities: Secondary outcomes of the ‘Physical Activity 4 Everyone’RCT. *International Journal of Obesity*, *40*(10), 1486-1493.

Sutherland, R., Campbell, E., Lubans, D. R., Morgan, P. J., Okely, A. D., Nathan, N., ... & Wiggers, J. (2013). A cluster randomised trial of a school-based intervention to prevent decline in adolescent physical activity levels: study protocol for the ‘Physical Activity 4 Everyone’trial. *BMC Public Health*, *13*(1), 1-10.

Sutherland, R., Campbell, E., Lubans, D. R., Morgan, P. J., Okely, A. D., Nathan, N., ... & Wiggers, J. (2016). ‘Physical Activity 4 Everyone’school-based intervention to prevent decline in adolescent physical activity levels: 12 month (mid-intervention) report on a cluster randomised trial. *British journal of sports medicine*, *50*(8), 488-495.

Sutherland, R. L., Campbell, E. M., Lubans, D. R., Morgan, P. J., Nathan, N. K., Wolfenden, L., ... & Williams, A. J. (2016). The physical activity 4 everyone cluster randomized trial: 2-year outcomes of a school physical activity intervention among adolescents. *American journal of preventive medicine*, *51*(2), 195-205.

<https://www.anzctr.org.au/Trial/Registration/TrialReview.aspx?id=362315>

**Janssen et al., PLAYgrounds**

**References:**

Janssen, M., Twisk, J. W., Toussaint, H. M., van Mechelen, W., & Verhagen, E. A. (2015). Effectiveness of the PLAYgrounds programme on PA levels during recess in 6-year-old to 12-year-old children. *British journal of sports medicine*, *49*(4), 259-264.

Janssen, M., Toussaint, H. M., Van Willem, M., & Verhagen, E. A. (2011). PLAYgrounds: Effect of a PE playground program in primary schools on PA levels during recess in 6 to 12 year old children. Design of a prospective controlled trial. *BMC public health*, *11*(1), 1-6.

<https://www.trialregister.nl/trial/2260>

<https://www.nji.nl/nl/Databank/Databank-Effectieve-Jeugdinterventies/Erkende-interventies/PLAYgrounds>

[www.playgrounds.nu](http://www.playgrounds.nu)

**Kelly et al., COPE TEEN**

**References:**

Kelly, S. A., Oswalt, K., Melnyk, B. M., & Jacobson, D. (2015). Comparison of intervention fidelity between COPE TEEN and an attention-control program in a randomized controlled trial. *Health Education Research*, *30*(2), 233-247.

Melnyk, B. M., Kelly, S., Jacobson, D., Belyea, M., Shaibi, G., Small, L., ... & Marsiglia, F. F. (2013). The COPE healthy lifestyles TEEN randomized controlled trial with culturally diverse high school adolescents: baseline characteristics and methods. *Contemporary clinical trials*, *36*(1), 41-53.

**Kennedy et al., Resistance Training for Teens**

**References**

Kennedy, S. G., Smith, J. J., Morgan, P. J., Peralta, L. R., Hilland, T. A., Eather, N., ... & Dewar, D. L. (2018). Implementing resistance training in secondary schools: a cluster randomized controlled trial. *Medicine & Science in Sports & Exercise*, *50*(1), 62-72.

Kennedy, S. G., Peralta, L. R., Lubans, D. R., Foweather, L., & Smith, J. J. (2019). Implementing a school-based physical activity program: process evaluation and impact on teachers’ confidence, perceived barriers and self-perceptions. *Physical Education and Sport Pedagogy*, *24*(3), 233-248.

Lubans, D. R., Smith, J. J., Peralta, L. R., Plotnikoff, R. C., Okely, A. D., Salmon, J., ... & Hilland, T. A. (2016). A school-based intervention incorporating smartphone technology to improve health-related fitness among adolescents: rationale and study protocol for the NEAT and ATLAS 2.0 cluster randomised controlled trial and dissemination study. *BMJ open*, *6*(6).

<https://www.anzctr.org.au/Trial/Registration/TrialReview.aspx?id=368026>

**Köykkä et al., Let’s Move It**

**References:**

Köykkä, K., Absetz, P., Araújo-Soares, V., Knittle, K., Sniehotta, F. F., & Hankonen, N. (2019). Combining the reasoned action approach and habit formation to reduce sitting time in classrooms: Outcome and process evaluation of the Let's Move It teacher intervention. *Journal of Experimental Social Psychology*, *81*, 27-38.

Hankonen, N., Heino, M. T., Araujo-Soares, V., Sniehotta, F. F., Sund, R., Vasankari, T., ... & HaUnited Kingdomkala, A. (2016). ‘Let’s Move It’-a school-based multilevel intervention to increase physical activity and reduce sedentary behaviour among older adolescents in vocational secondary schools: a study protocol for a cluster-randomised trial. *BMC Public Health*, *16*(1), 451.

<https://osf.io/v94fw>

<http://www.isrctn.com/ISRCTN10979479>

**Kien et al., Bewegte Klasse**

**References:**

Kien, C., Grillich, L., Nussbaumer-Streit, B., & Schoberberger, R. (2018). Pathways leading to success and non-success: a process evaluation of a cluster randomized physical activity health promotion program applying fuzzy-set qualitative comparative analysis. *BMC public health*, *18*(1), 1386.

Grillich, L., Kien, C., Takuya, Y., Weber, M., & Gartlehner, G. (2016). Effectiveness evaluation of a health promotion programme in primary schools: a cluster randomised controlled trial. *BMC public health*, *16*(1), 679.

<https://www.drks.de/drks_web/navigate.do?navigationId=trial.HTML&TRIAL_ID=DRKS00000622>

**Kocken et al., EF!**

**References:**

Kocken, P. L., Scholten, A. M., Westhoff, E., De Kok, B. P., Taal, E. M., & Goldbohm, R. A. (2016). Effects of a theory-based education program to prevent overweightness in primary school children. *Nutrients*, *8*(1), 12.

**Lonsdale et al., AMPED**

**References:**

Lonsdale, C., Lester, A., Owen, K. B., White, R. L., Peralta, L., Kirwan, M., ... & Kolt, G. S. (2019). An internet-supported school physical activity intervention in low socioeconomic status communities: results from the Activity and Motivation in Physical Education (AMPED) cluster randomised controlled trial. *British Journal of Sports Medicine*, *53*(6), 341-347.

Lonsdale, C., Lester, A., Owen, K. B., White, R. L., Moyes, I., Peralta, L., ... & Kolt, G. S. (2015). An internet-supported physical activity intervention delivered in secondary schools located in low socio-economic status communities: study protocol for the activity and motivation in physical education (AMPED) cluster randomized controlled trial. *BMC Public Health*, *16*(1), 17.

Lubans, D. R., Beauchamp, M. R., Diallo, T. M., Peralta, L. R., Bennie, A., White, R. L., ... & Lonsdale, C. (2018). School physical activity intervention effect on adolescents’ performance in mathematics. *Med Sci Sports Exerc*, *50*(12), 2442-50.

Peralta, L. R., Bennie, A., Gore, J., & Lonsdale, C. (2020). An Investigation of the Influence of Video Types and External Facilitation on PE Inservice Teachers’ Reflections and Their Perceptions of Learning: Findings From the AMPED Cluster Controlled Trial. *Journal of Teacher Education*, 0022487120964079.

<https://www.anzctr.org.au/Trial/Registration/TrialReview.aspx?id=365660>

**Lubans et al., ATLAS**

**References:**

Lubans, D. R., Smith, J. J., Plotnikoff, R. C., Dally, K. A., Okely, A. D., Salmon, J., & Morgan, P. J. (2016). Assessing the sustained impact of a school-based obesity prevention program for adolescent boys: the ATLAS cluster randomized controlled trial. *International Journal of Behavioral Nutrition and Physical Activity*, *13*(1), 92.

Smith, J. J., Morgan, P. J., Plotnikoff, R. C., Dally, K. A., Salmon, J., Okely, A. D., ... & Lubans, D. R. (2014). Rationale and study protocol for the ‘Active Teen Leaders Avoiding Screen-time’(ATLAS) group randomized controlled trial: an obesity prevention intervention for adolescent boys from schools in low-income communities. *Contemporary Clinical Trials*, *37*(1), 106-119.

Smith, J. J., Morgan, P. J., Plotnikoff, R. C., Dally, K. A., Salmon, J., Okely, A. D., ... & Lubans, D. R. (2014). Smart-phone obesity prevention trial for adolescent boys in low-income communities: the ATLAS RCT. *Pediatrics*, *134*(3), e723-e731.

Smith, J. J., Morgan, P. J., Plotnikoff, R. C., Stodden, D. F., & Lubans, D. R. (2016). Mediating effects of resistance training skill competency on health-related fitness and physical activity: The ATLAS cluster randomised controlled trial. *Journal of sports sciences*, *34*(8), 772-779.

<https://www.anzctr.org.au/Trial/Registration/TrialReview.aspx?id=363015&isReview=true>

**Martin et al., Active Classrooms**

**References:**

Martin, R., & Murtagh, E. (2017). Active classrooms: a cluster randomized controlled trial evaluating the effects of a movement integration intervention on the physical activity levels of primary school children. *Journal of physical activity and health*, *14*(4), 290-300.

Martin, R., & Murtagh, E. M. (2015). An intervention to improve the physical activity levels of children: design and rationale of the ‘Active Classrooms’ cluster randomised controlled trial. *Contemporary clinical trials*, *41*, 180-191.

<http://www.isrctn.com/ISRCTN14265493>

**McKay et al., AS! BC**

**References:**

McKay, H. A., Macdonald, H. M., Nettlefold, L., Masse, L. C., Day, M., & Naylor, P. J. (2015). Action Schools! BC implementation: from efficacy to effectiveness to scale-up. *British journal of sports medicine*, *49*(4), 210-218.

Naylor, P. J., Macdonald, H. M., Reed, K. E., & McKay, H. A. (2006). Action Schools! BC: a socioecological approach to modifying chronic disease risk factors in elementary school children.

Naylor, P. J., Macdonald, H. M., Zebedee, J. Reed, K. E., & McKay, H. A. (2006). Lessons learned from Action Schools! BC - an ‘active school’ model to promote physical activity in elementary schools. Journal of Science and Medicine in Sport 9:413-423

**Miller et al., PLUNGE**

**References:**

Miller, A., Christensen, E. M., Eather, N., Sproule, J., Annis-Brown, L., & Lubans, D. R. (2015). The PLUNGE randomized controlled trial: Evaluation of a games-based physical activity professional learning program in primary school physical education. *Preventive medicine*, *74*, 1-8.

Miller, A., Eather, N., Gray, S., Sproule, J., Williams, C., Gore, J., & Lubans, D. (2017). Can continuing professional development utilizing a game-centred approach improve the quality of physical education teaching delivered by generalist primary school teachers?. *European Physical Education Review*, *23*(2), 171-195.

<https://www.anzctr.org.au/Trial/Registration/TrialReview.aspx?id=364311>

**Morris et al., Unnamed**

**References:**

Morris, J. L., Daly-Smith, A., Defeyter, M. A., McKenna, J., Zwolinsky, S., Lloyd, S., ... & Graham, P. L. (2019). A Pedometer-Based Physically Active Learning Intervention: The Importance of Using Preintervention Physical Activity Categories to Assess Effectiveness. *Pediatric exercise science*, *31*(3), 356-362.

**Nader et al., BEPA**

**References:**

Abi Nader, P., Hilberg, E., Schuna Jr, J. M., John, D. H., & Gunter, K. B. (2019). Association of Teacher‐Level Factors With Implementation of Classroom‐Based Physical Activity Breaks. *Journal of School Health*, *89*(6), 435-443.

**Norris et al., Virtual Traveller**

**References:**

Norris, E., Dunsmuir, S., DUnited Kingdome-Williams, O., Stamatakis, E., & Shelton, N. (2018). Physically active lessons improve lesson activity and on-task behavior: A cluster-randomized controlled trial of the “Virtual Traveller” Intervention. *Health Education & Behavior*, *45*(6), 945-956.

Norris, E., Dunsmuir, S., DUnited Kingdome-Williams, O., Stamatakis, E., & Shelton, N. (2016). Protocol for the ‘Virtual Traveller’cluster-randomised controlled trial: a behaviour change intervention to increase physical activity in primary-school Maths and English lessons. *BMJ open*, *6*(6), e011982.

Norris, E., Dunsmuir, S., DUnited Kingdome-Williams, O., Stamatakis, E., & Shelton, N. (2018). Mixed method evaluation of the Virtual Traveller physically active lesson intervention: An analysis using the RE-AIM framework. *Evaluation and program planning*, *70*, 107-114.

**O’Leary et al., Project Spraoi**

O’Leary, M., Rush, E., Lacey, S., Burns, C., & Coppinger, T. (2019). Project Spraoi: two year outcomes of a whole school physical activity and nutrition intervention using the RE-AIM framework. *Irish Educational Studies*, *38*(2), 219-243.

Coppinger, T., Lacey, S., O'Neill, C., & Burns, C. (2016). ‘Project Spraoi’: A randomized control trial to improve nutrition and physical activity in school children. *Contemporary Clinical Trials Communications*, *3*, 94-101.

<http://www.isrctn.com/ISRCTN92611015>

**O'Neill et al., Michigan Model for Health**

**References:**

O'Neill, J. M., Clark, J. K., & Jones, J. A. (2016). Promoting fitness and safety in elementary students: a randomized control study of the Michigan model for health. *Journal of school health*, *86*(7), 516-525.

**Okely et al., Girls in Sport**

**References:**

Okely, A. D., Lubans, D. R., Morgan, P. J., Cotton, W., Peralta, L., Miller, J., ... & Janssen, X. (2017). Promoting physical activity among adolescent girls: the girls in sport group randomized trial. *International Journal of behavioral nutrition and physical activity*, *14*(1), 81.

Okely, A. D., Cotton, W. G., Lubans, D. R., Morgan, P. J., Puglisi, L., Miller, J., ... & Perry, J. (2011). A school-based intervention to promote physical activity among adolescent girls: rationale, design, and baseline data from the girls in sport group randomised controlled trial. *BMC public health*, *11*(1), 1-11.

Okely, A. D., Wright, J., Puglisi, L., Batterham., M., Janssen., X., Lubans, D. R., Morgan, P. J., Cotton, W. G., Peralta, L., Perry., J. (2012). Girls in Sport Intervention and Research Project. *Summary Report for the NSW Department of Education and Communities prepared by the Research Consortium.*

<http://www.anzctr.org.au/Trial/Registration/TrialReview.aspx?id=336305&isReview=true>

**Riley et al., E.A.S.Y. Minds**

**References:**

Riley, N., Lubans, D. R., Holmes, K., & Morgan, P. J. (2016). Findings from the EASY minds cluster randomized controlled trial: evaluation of a physical activity integration program for mathematics in primary schools. *Journal of Physical Activity and Health*, *13*(2), 198-206.

Riley, N., Lubans, D. R., Holmes, K., & Morgan, P. J. (2014). Rationale and study protocol of the EASY Minds (Encouraging Activity to Stimulate Young Minds) program: cluster randomized controlled trial of a primary school-based physical activity integration program for mathematics. *BMC Public Health*, *14*(1), 816.

Mavilidi, M. F., Lubans, D. R., Miller, A., Eather, N., Morgan, P. J., Lonsdale, C., . . . Riley, N. (2020). Impact of the “Thinking while Moving in English” intervention on primary school children's academic outcomes and physical activity: A cluster randomised controlled trial. International Journal of Educational Research, 102. doi:[10.1016/j.ijer.2020.101592](http://doi.org/10.1016/j.ijer.2020.101592)

<https://www.anzctr.org.au/Trial/Registration/TrialReview.aspx?id=364344>

**Robertson et al., FitQuest**

**References:**

Robertson, J., Macvean, A., Fawkner, S., Baker, G., & Jepson, R. G. (2018). Savouring our mistakes: Learning from the FitQuest project. *International journal of child-computer interaction*, *16*, 55-67.

<http://www.isrctn.com/ISRCTN11693550>

<https://judyrobertson.typepad.com/judy_robertson/data-for-exergame-for-childrens-activity-rct-and-qualitative-study-paper.html>

**Seibert et al., Unnamed**

**References:**

Seibert, T., Allen, D. B., Eickhoff, J. C., & Carrel, A. L. (2019). US Centers for Disease Control and Prevention‐Based Physical Activity Recommendations Do Not Improve Fitness in Real‐World Settings. *Journal of school health*, *89*(3), 159-164.

<https://clinicaltrials.gov/ct2/show/NCT02411552?id=NCT02411552&draw=2&rank=1>

**Smedegaard et al., Move for Wellbeing in School**

**References:**

Smedegaard, S., Brondeel, R., Christiansen, L. B., & Skovgaard, T. (2017). What happened in the ‘Move for Well-being in School’: a process evaluation of a cluster randomized physical activity intervention using the RE-AIM framework. *International Journal of Behavioral Nutrition and Physical Activity*, *14*(1), 159.

Smedegaard, S., Christiansen, L. B., Lund-Cramer, P., Bredahl, T., & Skovgaard, T. (2016). Improving the well-being of children and youths: a randomized multicomponent, school-based, physical activity intervention. *BMC Public Health*, *16*(1), 1127.

<http://www.isrctn.com/ISRCTN12496336>

**Sutherland et al., Unnamed**

**References:**

Sutherland, R. L., Nathan, N. K., Lubans, D. R., Cohen, K., Davies, L. J., Desmet, C., ... & Wolfenden, L. (2017). An RCT to facilitate implementation of school practices known to increase physical activity. *American Journal of Preventive Medicine*, *53*(6), 818-828.

<https://www.anzctr.org.au/Trial/Registration/TrialReview.aspx?id=368420>

**Tarp et al., LCoMotion**

**References:**

Tarp, J., Domazet, S. L., Froberg, K., Hillman, C. H., Andersen, L. B., & Bugge, A. (2016). Effectiveness of a school-based physical activity intervention on cognitive performance in Danish adolescents: lcomotion—learning, cognition and motion-a cluster randomized controlled trial. *PloS one*, *11*(6), e0158087.

Bugge, A., Tarp, J., Østergaard, L., Domazet, S. L., Andersen, L. B., & Froberg, K. (2014). LCoMotion-Learning, Cognition and Motion; a multicomponent cluster randomized school-based intervention aimed at increasing learning and cognition-rationale, design and methods. *BMC public health*, *14*(1), 1-8.

<https://clinicaltrials.gov/ct2/show/NCT02012881?term=NCT02012881&draw=2&rank=1>

**Tymms et al., the MOVE Project**

**References:**

Tymms, P. B., Curtis, S. E., Routen, A. C., Thomson, K. H., Bolden, D. S., Bock, S., ... & Summerbell, C. D. (2016). Clustered randomised controlled trial of two education interventions designed to increase physical activity and well-being of secondary school students: the MOVE Project. *BMJ open*, *6*(1).

<http://www.isrctn.com/ISRCTN82956355>

[http://www.move-project.org.United Kingdom/](http://www.move-project.org.uk/)

**van den Berg et al., Unnamed**

**References:**

van den Berg, V., Singh, A. S., Komen, A., Hazelebach, C., van Hilvoorde, I., & Chinapaw, M. J. (2019). Integrating juggling with math lessons: A randomized controlled trial assessing effects of physically active learning on maths performance and enjoyment in primary school children. *International journal of environmental research and public health*, *16*(14), 2452.

**Verloigne et al., Unnamed**

**References:**

Verloigne, M., Ridgers, N. D., De Bourdeaudhuij, I., & Cardon, G. (2018). Effect and process evaluation of implementing standing desks in primary and secondary schools in Belgium: a cluster-randomised controlled trial. *International Journal of Behavioral Nutrition and Physical Activity*, *15*(1), 94.

<https://clinicaltrials.gov/ct2/show/NCT03163004?term=standing+desks&cntry=BE&rank=1>

**Vik et al., UP4FUN**

**References:**

Vik, F. N., Lien, N., Berntsen, S., De Bourdeaudhuij, I., Grillenberger, M., Manios, Y., ... & Bere, E. (2015). Evaluation of the UP4FUN intervention: a cluster randomized trial to reduce and break up sitting time in European 10-12-year-old children. *PloS one*, *10*(3), e0122612.

Nanna Lien, Frøydis N. Vik, Sveinung Berntsen, Johannes Brug, Ilse De Bourdeaudhuij, Mai JM Chinapaw, Monika Grillenberger, Éva Kovács, Lea Maes, yannis manios, George Moschonis, Maartje M. van Stralen, Corinna Willhöft, Elling Bere: UP4FUN A school-based and family-involved intervention to reduce and break up sitting time among European 10-12 year olds - systematic development and formative evaluation. 04/2014; Universitetet i Agder. (Working Paper 165). http://hdl.handle.net/11250/194424., ISBN: 1504-9280

<http://www.isrctn.com/ISRCTN34562078>

**Wright et al., FLEX**

**References:**

Wright, C. M., Chomitz, V. R., Duquesnay, P. J., Amin, S. A., Economos, C. D., & Sacheck, J. M. (2019). The FLEX study school-based physical activity programs-measurement and evaluation of implementation. *BMC public health*, *19*(1), 73.

Wright, C. M., Duquesnay, P. J., Anzman-Frasca, S., Chomitz, V. R., Chui, K., Economos, C. D., ... & Sacheck, J. M. (2016). Study protocol: the Fueling Learning through Exercise (FLEX) study-a randomized controlled trial of the impact of school-based physical activity programs on children’s physical activity, cognitive function, and academic achievement. *BMC Public Health*, *16*(1), 1-12.

<https://clinicaltrials.gov/ct2/show/NCT02810834>

**Zhou et al., Chinese CHAMPS**

**References:**

Zhou, Z., Li, S., Yin, J., Fu, Q., Ren, H., Jin, T., ... & Yin, Z. (2019). Impact on Physical Fitness of the Chinese CHAMPS: A Clustered Randomized Controlled Trial. *International journal of environmental research and public health*, *16*(22), 4412.

Zhou, Z., Dong, S., Yin, J., Fu, Q., Ren, H., & Yin, Z. (2018). Improving physical fitness and cognitive functions in middle school students: Study protocol for the Chinese childhood health, activity and motor performance study (Chinese CHAMPS). *International journal of environmental research and public health*, *15*(5), 976.
