# Supplementary material for: Incomplete reporting of complex interventions: a call to action for journal editors to review their submission guidelines
Source: Trials. 2023 Mar 22;24:176. doi: 10.1186/s13063-023-07215-1 (PMC10031932; doi:10.1186/s13063-023-07215-1)
Supplement: Supplementary file 2 — Additional file 2. List of TIDieR items reported before and after author contact. [file 13063_2023_7215_MOESM2_ESM.docx]

**Additional file 2:** List of TIDieR checklist-based items reported before and after author contact

| **TIDieR checklist-based item [1]** | **Following**  **data extraction**  **(N=53)** | | | **Following author contact period**  **(N=53)** | | |
| --- | --- | --- | --- | --- | --- | --- |
|  | Available from majority? | N^a^ | % | Available from majority? | N^a^ | % |
| **Name of the intervention** | Yes | 53 | 100.0 | Yes | 53 | 100.0 |
| **Describe any rationale/theory** that underpinned the design of the teacher training provided (e.g. the Theory of Planned Behaviour). | No | 14 | 28.3 | Yes | 43 | 81.1 |
| **Materials:** Describe any physical or informational materials provided to teachers. | Yes | 38 | 71.7 | Yes | 48 | 90.6 |
| **Procedures:** Describe the procedures, activities, and/or processes used in the training, including any enabling or support activities. | Yes | 53 | 100.0 | Yes | 53 | 100.0 |
| **Who** provided the training? | Yes | 43 | 81.1 | Yes | 49 | 92.5 |
| **Mode** of delivery? (e.g. face-to-face, online, telephone, or multiple formats?) | Yes | 46 | 86.8 | Yes | 51 | 96.2 |
| Was the training delivered to staff **individually or in a group?** | No | 21 | 39.6 | Yes | 43 | 81.1 |
| **Where** was the training delivered? (describe the location and any necessary infrastructure) | No | 20 | 37.7 | Yes | 44 | 83.0 |
| **Total mins/hours** of training? | Yes | 38 | 71.7 | Yes | 47 | 88.7 |
| **How many** training **sessions** were provided? | Yes | 42 | 79.2 | Yes | 50 | 94.3 |
| **Over what time period?** | Yes | 37 | 69.8 | Yes | 47 | 88.7 |
| Was the training planned to be **personalised**? (e.g. was the training tailored based on teachers’ skills?) | No | 20 | 37.7 | Yes | 45 | 84.9 |
| Was the training **modified** during the intervention? (i.e. any unforeseen modifications made to the overall training provided? e.g. teachers did not receive paper manuals as planned) | No | 3 | 5.7 | Yes | 38 | 71.7 |
| Was **adherence or fidelity** to the planned teacher training assessed? Were any strategies used to maintain training fidelity? If so, describe any strategies, and the extent to which the teacher training was delivered as planned. | No | 3 | 5.7 | Yes | 40 | 75.5 |
| Was **attendance of teachers monitored** at training? If so, describe any outcomes. | No | 14 | 26.4 | Yes | 44 | 83.0 |

^a^ Item was identified as present or not applicable

1. Hoffmann TC, Glasziou PP, Boutron I, Milne R, Perera R, Moher D, Altman DG, Barbour V, Macdonald H, Johnston M *et al*. Better reporting of interventions: template for intervention description and replication (TIDieR) checklist and guide. *BMJ* 2014, 348:g1687.
