# Supplementary material for: Incomplete reporting of complex interventions: a call to action for journal editors to review their submission guidelines
Source: Trials. 2023 Mar 22;24:176. doi: 10.1186/s13063-023-07215-1 (PMC10031932; doi:10.1186/s13063-023-07215-1)
Supplement: Supplementary file 3 — Additional file 3. Complete list of journals contacted (n=32) and action taken (n=7). [file 13063_2023_7215_MOESM3_ESM.docx]

**Additional file 3:** Complete list of journals contacted (n=32) and action taken* (n=7)

1. American Journal of Preventive Medicine
2. BMC Public Health
3. BMJ
4. BMJ Open
5. British Journal of Sports Medicine*
6. European Physical Education Review
7. Evaluation and Program Planning*
8. Health Education & Behavior
9. Health Education Research
10. Health Technology Assessment
11. International Journal of Child-Computer Interaction
12. International Journal of Environmental Research and Public Health
13. International Journal of Obesity
14. Irish Educational Studies
15. Journal of Experimental Social Psychology*
16. Journal of Paediatrics & Child Health
17. Journal of Physical Activity & Health*
18. The Journal of School Health
19. Journal of Science & Medicine in Sport*
20. Journal of Sports Sciences
21. Journal of Teaching in Physical Education
22. Medicine and Science in Sports and Exercise
23. Nutrients
24. Pediatric Exercise Science
25. Physical Education & Sport Pedagogy
26. PLoS ONE
27. Preventive Medicine
28. Preventive Medicine Reports
29. Psychology of Sport & Exercise*
30. Scandinavian Journal of Educational Research
31. Translational Journal of the American College of Sports Medicine
32. Sport, Exercise, and Performance Psychology*

* As of March 8th, 2023, journals marked with an asterisk (*) reported changes made to their submission guidelines. With the exception of 'Sport, Exercise, and Performance Psychology', who we contacted in July 2022, all other journals were initially contacted in May 2022.
